# Experimental observation of gain in a resonantly pumped Pr^3+^-doped chalcogenide glass mid-infrared fibre amplifier notwithstanding the signal excited-state absorption

**DOI:** 10.1038/s41598-019-47432-w

**Published:** 2019-08-06

**Authors:** Meili Shen, David Furniss, Mark Farries, Dinuka Jayasuriya, Zhuoqi Tang, Lukasz Sojka, Slawomir Sujecki, Trevor M. Benson, Angela B. Seddon

**Affiliations:** 1 Mid-Infrared Photonics Group, George Green Institute for Electromagnetics Research, Faculty of Engineering, University Park, University of Nottingham, Nottingham, NG7 2RD UK; 2 Telecommunications and Teleinformatics Department, Wroclaw University of Technology, Wybrzeze Wyspianskiego 27, 50-370 Wroclaw, Poland

**Keywords:** Fibre lasers, Fibre lasers, Fibre lasers, Fibre lasers, Fibre lasers

## Abstract

We demonstrate a maximum gain of 4.6 dB at a signal wavelength of 5.28 μm in a 4.1 μm resonantly pumped Pr^3+^-doped selenide-based chalcogenide glass fibre amplifier of length 109 mm, as well as a new signal excited-stated absorption (ESA) at signal wavelengths around 5.5 μm. This work to the best of our knowledge is the first experimental demonstration of gain at mid-infrared (MIR) wavelengths in a Pr^3+^-doped chalcogenide fibre amplifier. The signal ESA of the fibre is attributed to the transition ^3^H_6_ → (^3^F_4_, ^3^F_3_) after the pump ESA (^3^H_5_ → ^3^H_6_) at a pump wavelength of 4.1 μm, which absorbs the MIR signal at wavelengths of 5.37, 5.51 and 5.57 μm, and so spoils the amplifier’s performance at these wavelengths. Thus, this signal ESA should be suppressed in a resonantly pumped Pr^3+^-doped selenide-based chalcogenide fibre amplifier.

## Introduction

Mid-infrared (MIR) laser sources are of great demand in the fields of biomedical sensing, environmental monitoring and free space communication^1^. Particularly, the 3–12 μm wavelength range has attracted significant interest within the MIR region because it covers two of the transparent atmospheric windows, as well as the vibrational resonant bands of several chemical bonds, such as [S-O], [C-H], and [C=O]. Particularly, the MIR spectral region at 6 µm and beyond is important for accessing the fingerprint region of molecular vibration bands for cancer diagnostics^2,3^. Broadband emission can be achieved by supercontinuum generation, but this required a pump laser with a wavelength >4.5 µm^4,5^. Hence, high brightness, high efficiency, and compact MIR fibre lasers aiming for longer wavelengths are promising sources for the growing needs of these advanced applications.

Recently, rare-earth (RE) ion doped fluoride fibres has achieved high output power MIR fibre lasers with tuneable wavelength. In particular, an Er^3+^-doped fluoride fibre has shown 2.6–3 μm lasing with several tens of Watts’ power^6^. Moreover, 3.55 μm lasing wavelength has been reported by dual-wavelength pumping an Er^3+^-doped fluoride fibre^7^. Versatile laser operations in the MIR wavelength range of 2.8–3.92 μm have also been provided by Ho^3+^ and Dy^3+^ doped fluoride fibres^8–12^. As the modified indium fluoride (InF_3_) host glass has been developed, ~4.2 μm fluorescence has been first demonstrated in the Dy^3+^-doped InF_3_ fibre^13^. Although the new InF_3_ host glass has further reduced the phonon energy to 509 cm^−1^^14^, it remains relatively high for laser operation at wavelengths beyond 4 μm, which becomes the main challenge of the RE ion doped fluoride glass fibre lasers.

Due to their lower phonon energy, of ~300 cm^−1^, RE ion doped chalcogenide glass fibres have been investigated as promising candidates for longer wavelength MIR lasers^2^. Intensive 3–8 μm photoluminescence (PL) has been observed in Pr^3+^, Dy^3+^, Tb^3+^ and Sm^3+^-doped chalcogenide glass fibres^15–20^. Besides, optical amplification at 1.08 μm with maximum gain of 6.8 dB has been demonstrated in a neodymium-doped chalcogenide glass fibre amplifier^21^.

Among them, Pr^3+^-doped chalcogenide glass fibre has been fabricated into small-core step index fibre with optical fibre loss around 2 dB/m, which can be applied practically towards a MIR laser^22,23^. The transition of the Pr^3+^ ion from the first excited-state ^3^H_5_ to the ground-state ^3^H_4_ presents a broad emission cross-section from 4 to 6 μm, and the absorption cross-section within these two states is from 3.5 to 5.5 μm^24,25^. Thus, the transition ^3^H_5_ → ^3^H_4_ suggests that resonant pumping at wavelengths around 4 μm is accessible with Pr^3+^-doped chalcogenide fibre, and tends to achieve higher efficiency due to the diminished quantum defect between the pump and signal wavelengths. In our recent work, we have numerically investigated the resonantly pumped Pr^3+^-doped chalcogenide glass MIR fibre amplifier, which considered the influence of excited-state absorption (ESA) on the first excited-state ^3^H_5_^25^. The modelling results suggested that more than 62.8% power conversion efficiency could be achieved within the signal wavelength range of 4.5–5.3 μm using a resonantly pumped Pr^3+^-doped fibre amplifier.

An up-conversion process: ^3^H_6_ + ^3^H_6_ → ^3^F_3_ + ^3^H_5_ has been reported in the Pr^3+^: LaCl_3_ crystal^26^. The Pr^3+^ ions populating the ^3^H_6_ level were excited to the upper ^3^F_3_ level by the up-conversion process, which facilitated the 5.2 μm lasing due to the transition ^3^F_3_ → ^3^H_6_^26^. Accordingly, it is indicated that excited-stated absorption around 5.2 μm might present in the Pr^3+^ ions, originating from the transition ^3^H_6_ → ^3^F_3_. However, this transition may affect the amplification of signal wavelengths longer than 5 μm in the resonantly pumped Pr^3+^-doped chalcogenide fibre amplifier, and has not hitherto been reported in Pr^3+^-doped selenide-based chalcogenide fibre.

In this paper, we report a maximum gain of 4.6 dB at a signal wavelength of 5.28 μm in a 4.1 μm resonantly pumped Pr^3+^-doped chalcogenide selenide-based glass fibre amplifier. We believe this to be the first experimental demonstration of gain at MIR wavelengths in a Pr^3+^-doped chalcogenide fibre amplifier. We further report a new signal ESA in this fibre amplifier that will absorb the MIR signal at wavelengths of 5.37, 5.51, and 5.57 μm. Measurements of the output signal spectra at different pump powers and near-infrared (NIR) PL reveal the transitions within the energy levels of the Pr^3+^ ion associated with the signal ESA. The influence of the signal ESA around 5.5 μm on the signal amplification is discussed, as well as possible methods for suppressing the ESA.

## Amplifier Configuration

**Figure 1:**
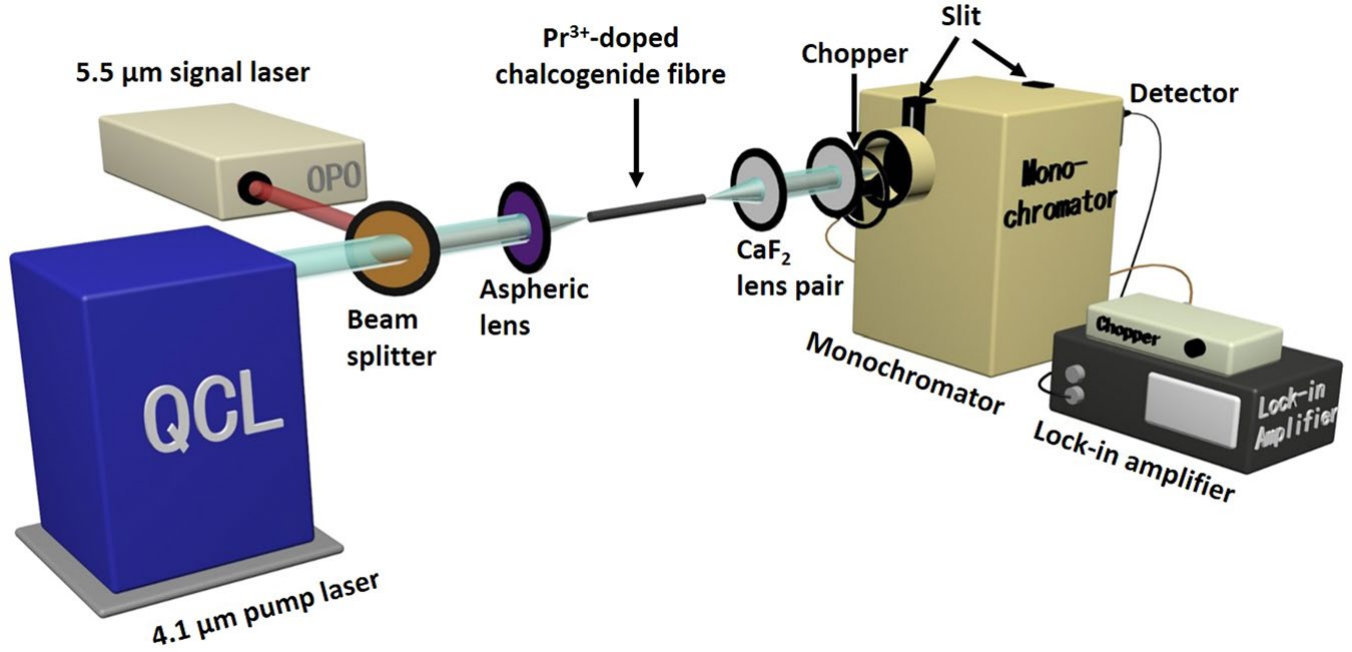
Experimental set-up of a 4.1 μm resonantly pumped 1000 ppmw Pr^3+^-doped selenide-based chalcogenide MIR fibre amplifier with a 5.5 μm signal laser (QCL: quantum cascade laser; OPO: optical parametric oscillator).

The experimental set-up of the resonantly pumped Pr^3+^-doped chalcogenide MIR fibre amplifier is depicted in Fig. 1. The pump laser was a 4.1 μm quantum cascade laser (QCL) (Pranalytica Inc.). The signal laser was provided by a pulsed optical parametric oscillator (OPO) (Chromacity Ltd.) with a repetition rate of 1 MHz and a pulse width of 1–2 ps. The actual spectrum of the signal laser was broad, spanning from 5.1 to 5.7 μm, and peak intensity was located at 5.5 μm. The average output power of the OPO was fixed at 40 mW. The pump and signal lasers were combined using a MIR beam splitter (Thorlabs Inc.), which had 60% reflectivity at 5.5 μm, and 40% transmission at 4.1 μm. The two laser beams were kept in a co-axis status after the beam splitter, and con-focused on the end of the active fibre using an aspheric lens with a focal length of 5.95 mm (Thorlabs Inc.), which allowed more than 90% signal and pump power to pass through. In this case, the maximum signal power launched into the fibre was about 20 mW after considering the power loss from the lenses and the Fresnel reflection around 20% at the input fibre face. The 4.1 μm pump power launched into the fibre was adjusted to vary from 0 to 120 mW. The Pr^3+^-doped chalcogenide fibre was placed in a three-staged V groove. The output beams from the fibre were collimated and focused on to the monochromator using a CaF_2_ lens pair with a 50 and a 40 mm focus length (Thorlabs Inc.), respectively. A chopper was placed between the latter CaF_2_ lens and the monochromator to modulate the output beam with the frequency of 70 Hz. The output spectrum from 5.1 μm to 5.7 μm was recorded using a MIR HgCdTe detector (3–6 μm, VIGO System S.A. Ltd.), which was connected to a lock-in amplifier (Brookdale Electronics Inc.). To avoid the output signal power exceeding the measurement range of the detector, two slits, each of width 0.25 mm, were placed over both the entrance and exit of the monochromator.

## Results and Discussion

### Fibre characterisation

**Figure 2:**
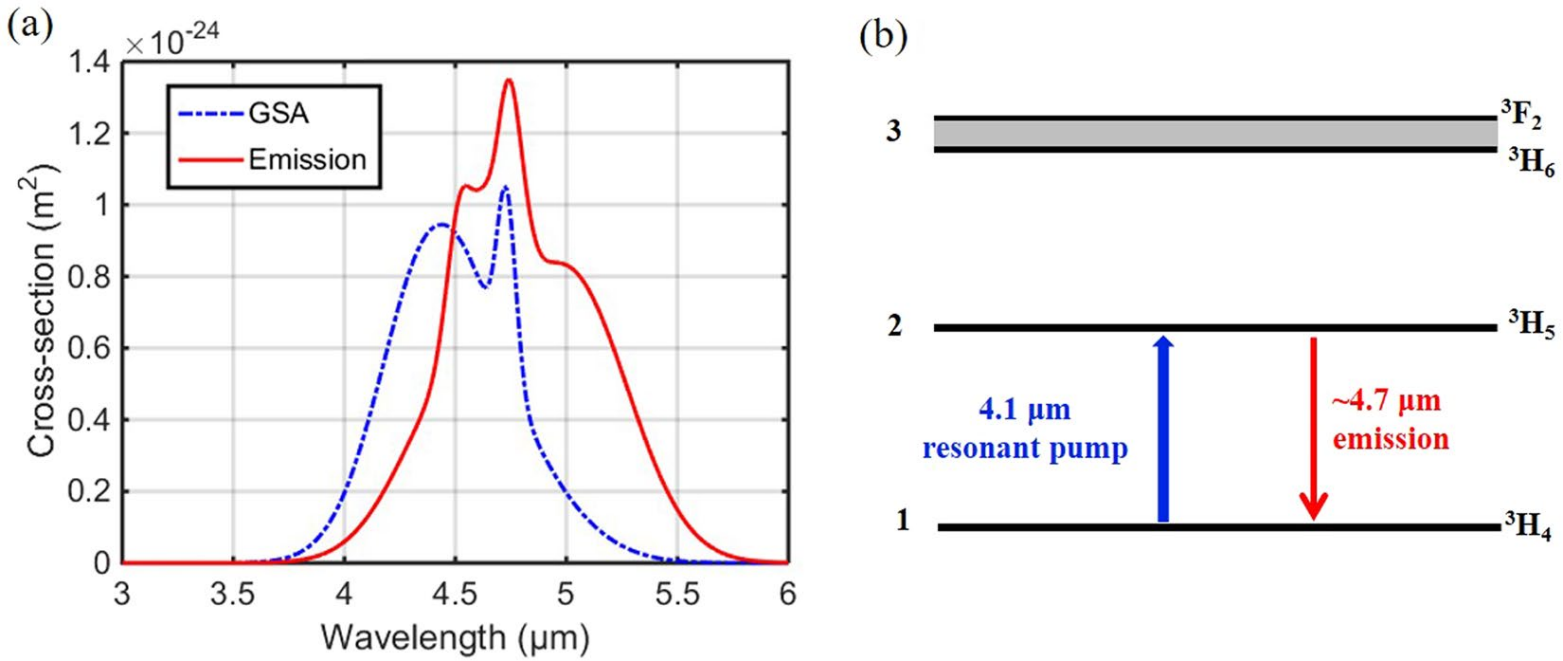
(**a**) Cross-section of the GSA due to the transition ^3^H_4_ → ^3^H_5_ and the emission cross-section of the transition ^3^H_5_ → ^3^H_4_. (**b**) Simplified energy diagram of Pr^3+^ ions in the 4.1 μm resonant pumping scheme.

The MIR PL of the Pr^3+^-doped chalcogenide fibre was first measured, before the resonantly pumped Pr^3+^-doped fibre amplifier was investigated. The fibre sample tested for MIR PL was a 50 mm long unstructured 1000 ppmw Pr^3+^-doped Ge-As-Se-Ga fibre with a core diameter of ~230 μm and no cladding. During the PL measurement, the OPO was switched off and only the 4.1 μm pump laser was launched into the fibre sample. The pump wavelength was positioned in the range of the ground-state absorption (GSA), as shown in Fig. 2(a). According to the simplified energy diagram of the Pr^3+^ ion presented in Fig. 2(b), the potential MIR amplification of the resonantly pumped Pr^3+^-doped chalcogenide fibre is mainly attributed to the transition ^3^H_5_ → ^3^H_4_. The emission cross-section (ECS) corresponding to this transition is shown in the red curve in Fig. 2(a); it covers the wavelength range from 3.5 to 6 μm. This indicates that the MIR PL spectrum generated from the resonantly pumped Pr^3+^-doped fibre should also be within this range.

**Figure 3:**
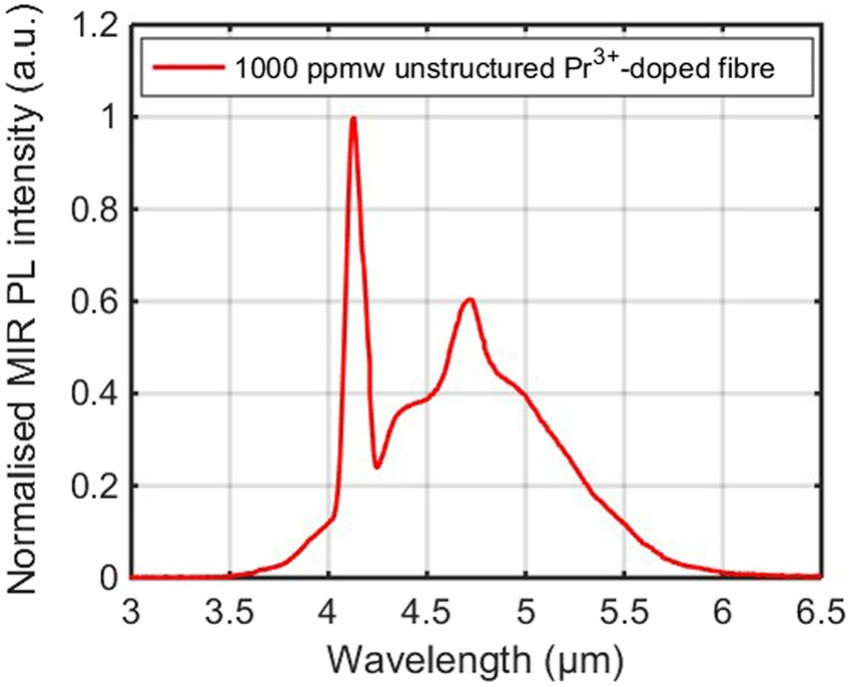
Normalised MIR PL spectrum from the 1000 ppmw Pr^3+^-doped Ge-As-Se-Ga fibre with the 4.1 μm pump laser operating at 65 mW.

Figure 3 presents the MIR PL spectrum of the Pr^3+^-doped Ge-As-Se-Ga fibre under the 4.1 μm resonant pumping with a pump power of 65 mW. As expected, the actual PL of the fibre sample spans from 3.5 to 6 μm and the highest peak intensity is at 4.7 μm, consistent with ECS shown in Fig. 2(a). The distinct peak at 4.1 μm presented in Fig. 3 is the residual pump laser. Most of the air along the output optical path had been purged in the experimental set-up to remove the influence of CO_2_ absorption at 4.2 μm. The small dip observed at 4.6 μm is attributed to Se-H contamination absorption in the fibre^27^. It should be noted that the spectrum presented in Fig. 3 was normalised to ‘1’ by dividing by the highest peak of the residual pump laser without the system correction. Nevertheless, the MIR PL spectrum spanning from 3.5–6 μm has been obtained in the 4.1 μm resonantly pumped unstructured 1000 ppmw Pr^3+^-doped selenide-based chalcogenide glass fibre, which clearly reveals the potential for MIR signal amplification using this Pr^3+^-doped chalcogenide fibre.

### Amplification and the signal ESA

Based on the above MIR PL measurement, the experiment for the resonantly pumped Pr^3+^-doped chalcogenide fibre amplifier was implemented. The OPO laser and QCL were both switched on to provide signal (~5.5 µm) and pump (4.1 µm) lasers for the fibre amplifier, respectively. The 1000 ppmw Pr^3+^-doped Ge-As-Se-Ga active fibre used for the signal amplification was the same batch of the fibre used in the above MIR PL measurement, except the length of the active fibre was increased from 50 mm to 109 mm.

**Figure 4:**
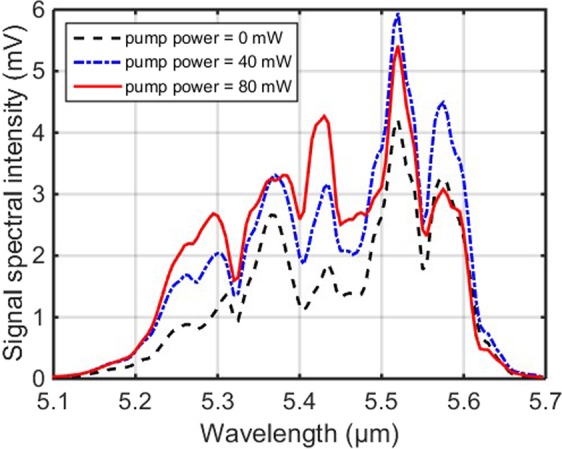
The output signal spectrum of the ~5.5 μm OPO laser at pump powers of 0, 40, and 80 mW, respectively, in the 4.1 μm resonantly pumped Pr^3+^-doped chalcogenide fibre amplifier.

The experimental results of the output signal spectrum of the resonantly pumped the Pr^3+^-doped fibre amplifier are presented in Fig. 4. As the 4.1 μm pump power was increased from 0 mW to 40 mW, the intensity of the entire signal spectrum was apparently increased, and the maximum gain of 3.1 dB was obtained around the wavelength of 5.3 μm. The increase of the signal spectrum intensity indicates that the MIR signal laser is successfully amplified in the resonantly pumped Pr^3+^-doped fibre amplifier, which is consistent with the numerical simulation in our previous work^25^. This is also the first time that the amplification of the MIR laser has been observed in the Pr^3+^-doped selenide-based chalcogenide fibre. For the resonant pumping scheme, Pr^3+^ ions can directly populate the first excited-state ^3^H_5_ and transit to the ground-state ^3^H_4_ with a strong PL emission over 3.5–6 μm, as presented in Fig. 3. Therefore, the signal wavelengths beyond 4 μm are more likely to be amplified in the resonantly pumped Pr^3+^-doped fibre amplifier attributed to the transition ^3^H_5_ → ^3^H_4_. Due to the lack of an appropriate dichroic mirror or a band pass filter, the output signal laser and residual pump laser of the amplifier could not be separated in our experiment. Thus, the output power and the efficiency of this fibre amplifier have not been characterised at present. This will be further investigated in our future work.

Subsequently, when the 4.1 μm pump power was increased to 80 mW, different evolution trends emerged for the output signal spectrum shown in Fig. 4. It was found that the signal spectral intensity was still amplified for signal wavelengths in the range of 5.2–5.48 μm. However, for signal wavelengths above 5.48 μm, the spectral intensity shows an obvious decrease compared to the situation of 40 mW pump power.

**Figure 5:**
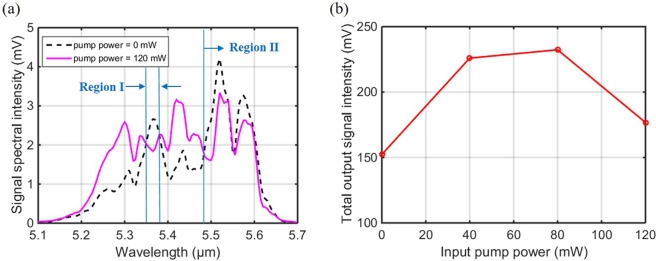
For the 4.1 μm resonantly pumped Pr^3+^-doped chalcogenide fibre amplifier: (**a**) the output signal spectrum of the ~5.5 μm OPO laser when the pump power was at 0 and 120 mW, respectively, and (**b**) the total output signal intensity when the pump power was varied from 0 to 120 mW.

The signal spectral intensity was further decreased in the wavelength region of signals longer than 5.48 μm when the pump power was lifted to 120 mW, as presented in Fig. 5(a). More importantly, it can be seen that for signal wavelengths within Region I (5.35–5.38 μm) and Region II (>5.48 μm), the signal spectral intensity at these wavelengths is now even lower than the initial spectral intensity obtained when the pump power is 0 mW. From the above discussion on the output signal spectrum, it is concluded that the spectral intensity at signal wavelengths less than 5.48 μm is favourably amplified when the 4.1 μm pump power was increased to 80 mW. However, with further increase of the pump power beyond 80 mW to 120 mW, then the spectral intensity to some extent shows a decline at signal wavelengths involved in Region I and II [see Fig. 5(a)]. To figure out the dependency of the total output signal intensity over the wavelength range 5.1–5.7 μm with increasing 4.1 μm pump power, the signal spectral intensity was integrated across the individual wavelengths to calculate the total output intensity. Figure 5(b) plots the total output signal intensity as a function of the input pump power. Similarly, an evident reduction is shown in the total output signal intensity, as the pump power was increased above 80 mW, which demonstrates that the ~5.5 μm signal power was likely to be absorbed rather than being successively amplified in the Pr^3+^-doped Ge-As-Se-Ga fibre with higher pump powers at 4.1 μm.

**Figure 6:**
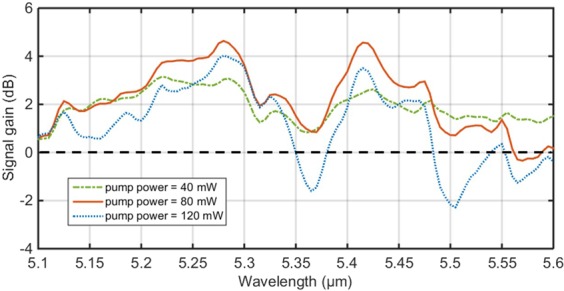
The signal gain spectrum at pump powers of 40, 80 and 120 mW, respectively, in the 4.1 μm resonantly pumped Pr^3+^-doped chalcogenide fibre amplifier.

To analyse better the decrease of the output signal intensity at higher pump powers, the signal gain spectrum was also calculated when the 4.1 μm pump power was successively changed to be 40, 80 and 120 mW, respectively, as shown in Fig. 6. It can be seen from Fig. 6 that the maximum gain of the amplifier is 4.6 dB at a signal wavelength of 5.28 μm for 80 mW pump power. However, three discrete negative peaks, indicating negative gain, were observed at signal wavelengths of 5.37 μm, 5.51 μm and 5.57 μm, as the pump power was increased to 120 mW. This observation suggests that the reason for the decrease of the signal intensity is signal absorption at these wavelengths; such absorption would greatly hamper the sustained growth of the output signal intensity with increasing pump power. It should be noted that, compared with the situation of 80 mW pump power, at 120 mW pump power the signal gain also decreased but remained higher than 0 dB at wavelengths shorter than 5.3 μm. This phenomenon might arise due to the stronger pump ESA excited by the higher pump power, then more pump power could be converted to the near-infrared (NIR) amplified spontaneous emission (ASE) rather than to the MIR signal amplification.

The dips of the negative signal gain shown at wavelengths of 5.37 μm, 5.51 μm and 5.57 μm represent that the signals are absorbed by the Pr^3+^ ions rather than being amplified. However, these wavelengths are not included in the main absorption wavelength ranges belonging to the transition ^3^H_4_ → ^3^H_5_ (GSA), nor the transition ^3^H_5_ → (^3^F_2_, ^3^H_6_) which we denote as the pump ESA in the Pr^3+^ ion. This suggests that a new signal ESA, associated with an even higher energy level, is triggered. Based on the literature, the emission wavelength of the transition ^3^F_3_ → ^3^H_6_ reported in the Pr^3+^: LaCl_3_ crystal laser was around 5.2 μm (~1907 cm^−1^) when the crystal temperature was fixed at 130 K^26^; this is close to the particularly absorbed signal wavelengths presented in Fig. 6. Therefore, the absorption of the signal at wavelengths: 5.37, 5.51 and 5.57 μm, we propose, is due to the transition ^3^H_6_ → (^3^F_4_, ^3^F_3_), that is the signal ESA in the Pr^3+^-doped selenide-based chalcogenide fibre. The three individual absorption wavelengths found in this signal ESA might be related with a complicated Stark splitting of the thermally coupled levels (^3^F_4_, ^3^F_3_)^26^.

**Figure 7:**
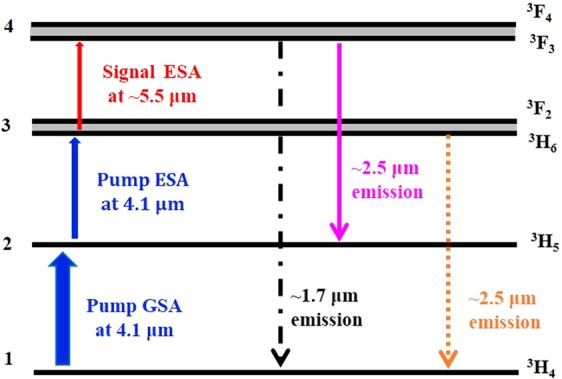
Simplified energy diagram of Pr^3+^ ions in the 4.1 μm resonant pumping scheme with the 5.5 μm signal laser. There are two potential downward transitions from the level (^3^F_4_, ^3^F_3_), with respective emissions at around 1.7 and 2.5 μm, and also the down transition (^3^F_2_, ^3^H_6_) → ^3^H_4_ exhibits emission around 2.5 μm.

For the 4.1 μm resonantly pumped Pr^3+^-doped chalcogenide fibre amplifier, the Pr^3+^ ions generally populating the energy level ^3^H_5_, after the GSA of a 4.1 μm pump photon, can be excited to the upper energy level (^3^F_2_, ^3^H_6_) by the pump ESA of another 4.1 μm pump photon, as shown in Fig. 7. In this case, two possible transitions could take place for the Pr^3+^ ions on the level (^3^F_2_, ^3^H_6_). On the one hand, they might transit to the ground state ^3^H_4_ and generate NIR fluorescence around 2.5 μm, as demonstrated in the literature^24,25,28^. On the other hand, these Pr^3+^ ions populating the level (^3^F_2_, ^3^H_6_) might be further excited to the higher energy level (^3^F_4_, ^3^F_3_) by the signal ESA of a photon with wavelength around 5.5 μm.

According to the energy diagram shown in Fig. 7, after the signal ESA at ~5.5 μm, the Pr^3+^ ions populating the energy level (^3^F_4_, ^3^F_3_) may transit to the energy level ^3^H_5_ with fluorescence near 2.5 μm, or transit to the ground state ^3^H_4_ with fluorescence near 1.7 μm^3,17,29^. Given that the signal ESA exists, the PL spectrum at 2.5 μm or 1.7 μm will change before and after the signal ESA taking place. In our experiment, NIR PL was measured by replacing the MIR HgCdTe detector with an extended InGaAs detector (1.8–2.6 μm, Thorlabs Inc.). Due to the wavelength sensitivity of the extended InGaAs detector, the measurement of 1.7 μm fluorescence emission from the level (^3^F_4_, ^3^F_3_) was unavailable. As a consequence, the NIR PL emission around 2.5 μm generated from the higher energy level (^3^F_4_, ^3^F_3_) alone is used to provide evidence for the signal ESA around the wavelength of 5.5 μm.

**Figure 8:**
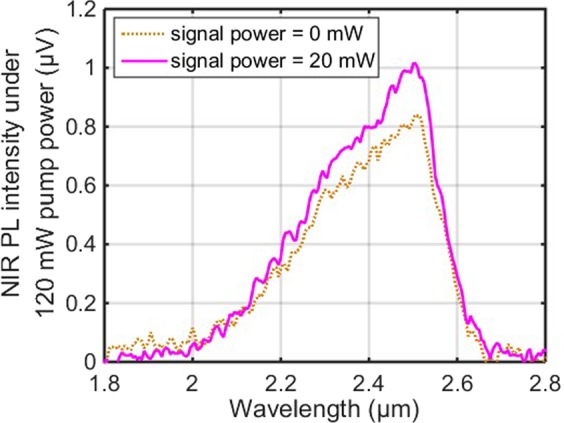
The NIR PL spectrum from the 4.1 μm resonantly pumped Pr^3+^-doped chalcogenide fibre amplifier when the ~5.5 μm signal power was at 0, and 20 mW, respectively. The 4.1 μm pump power was maintained at 120 mW.

The NIR PL around 2.5 μm of the resonantly pumped Pr^3+^-doped fibre amplifier was measured with and without the signal laser. Figure 8 presents the NIR PL spectrum when the signal laser is off (0 mW) and the 4.1 μm pump power is at 120 mW. It spans from 2 to 2.7 μm with the peak at 2.5 μm. This NIR emission is related to the transition (^3^F_2_, ^3^H_6_) → ^3^H_4_ of the Pr^3+^ ions due to the pump ESA of 4.1 μm pump laser, and has been previously reported in the resonantly pumped Pr^3+^-doped selenide chalcogenide fibre^24,25^. Under the same pump power (120 mW), the sufficient Pr^3+^ ions populating the level (^3^F_2_, ^3^H_6_) can be excited to the level (^3^F_4_, ^3^F_3_) by the signal laser around 5.5 μm. With a signal power of 20 mW, the spectral intensity at 2.5 μm was further increased compared with that when the signal power was off, which suggests that the additional NIR PL emission at ~2.5 μm originated from the transition (^3^F_4_, ^3^F_3_) → ^3^H_5_. This proves the existence of the signal ESA at ~5.5 μm, which is attributed to the transition ^3^H_6_ → (^3^F_4_, ^3^F_3_), in Pr^3+^-doped selenide chalcogenide fibre. To our best knowledge, it is the first time that the presence of the signal ESA around 5.5 μm has been observed in a resonantly pumped Pr^3+^-doped chalcogenide fibre amplifier.

The signal ESA at around 5.5 μm of the Pr^3+^ ions will tend to have been suppressed in the 4.1 μm resonantly pumped Pr^3+^-doped fibre amplifier due to the propensity for absorption of the signal laser to generate additional NIR ASE. A key approach to suppressing this undesirable signal ESA, is to reduce the ®pump ESA at 4.1 μm of the Pr^3+^ ion. One possible method to do this latter, is to increase the signal laser power, namely to saturate the fibre amplifier^30^, so that the majority of the Pr^3+^ ions populating the ^3^H_5_ level will transit down to the ground state rather than suffering pump ESA. A second method would be to optimise the signal wavelength so as to ensure it is not within the wavelength range of the most severe signal ESA. Last but not least, the pump wavelength could beneficially be shifted to longer than 4.5 μm, *i*.*e*. out of the absorption range of the pump ESA. As predicted in our recent modelling work, utilising a pump laser of wavelength longer than 4.5 μm would not only suppress the pump ESA but also, it is suggested, would achieve a high efficiency, resonantly pumped Pr^3+^-doped fibre amplifier^24^. Furthermore, to improve the performance of the MIR signal amplification, the current experimental set-up of the resonantly pumped Pr^3+^-doped chalcogenide fibre amplifier could be improved in the following aspects: (1) an appropriate MIR dichroic mirror should be placed at the output end of the fibre in order to separate the output signal laser from the residual pump laser, thereby to measure the amplified signal power and power conversion efficiency; (2) replace the active fibre with a small-core Pr^3+^-doped Ge-As-Se-Ga fibre of lower fibre loss over the wavelength range of 3.5–6 μm, as the gain medium.

## Conclusions

In conclusion, we demonstrated a maximum gain of 4.6 dB at a signal wavelength of 5.28 μm, in a 4.1 μm resonantly pumped Pr^3+^-doped chalcogenide selenide-based glass fibre amplifier of length 109 mm with a broadband signal centred on 5.5 μm, as well as a new signal ESA at around 5.5 μm. This work to the best of our knowledge is the first experimental demonstration of gain at MIR wavelengths and the first observation of the signal ESA from a Pr^3+^-doped selenide-based chalcogenide fibre. During the process of the signal ESA, the Pr^3+^ ions populating the (^3^F_2_, ^3^H_6_) level after the pump ESA at 4.1 um, were further excited to the higher energy level (^3^F_4_, ^3^F_3_) by absorbing the MIR signal around 5.5 μm. This process negatively affected the signal amplification, particularly at the wavelengths of 5.37, 5.51 and 5.57 μm. The suppression of the signal ESA might be achieved by increasing the saturation of the fibre amplifier, selecting a proper signal wavelength, or shifting the pump laser to beyond 4.5 μm in the resonantly pumped Pr^3+^-doped fibre amplifier.

## Data Availability

The dataset generated and analysed during the current study are available from the corresponding author on reasonable request.
